# Analysis of Chemical Composition and In Vitro and In Vivo Antifungal Activity of *Raphanus raphanistrum* Extracts against *Fusarium* and *Pythiaceae*, Affecting Apple and Peach Seedlings

**DOI:** 10.3390/molecules26092479

**Published:** 2021-04-23

**Authors:** Sabrine Mannai, Najwa Benfradj, Ahlem Karoui, Ibtissem Ben Salem, Amel Fathallah, Mahmoud M’Hamdi, Naima Boughalleb-M’Hamdi

**Affiliations:** Department of Biological Sciences and Plant Protection, High Institute of Agronomy of Chott Mariem, University of Sousse, UR13AGR03, 4042 Sousse, Tunisia; sabrinemannai@hotmail.com (S.M.); najwabenfradj@yahoo.fr (N.B.); ahlemkar2005@yahoo.fr (A.K.); ibtibesa82@gmail.com (I.B.S.); amel_fat@yahoo.fr (A.F.); mhamdimahmoud@yahoo.fr (M.M.)

**Keywords:** *Raphanus raphanistrum*, aqueous, organic extracts, liquid chromatography, gas chromatography, biocontrol, glucosinolates

## Abstract

The goal of this investigation was to evaluate the in vitro and in vivo efficiency of *Raphanus raphanistrum* extracts against *Fusarium* and *Pythiaceae* species associated with apple and peach seedling decline in Tunisian nurseries. A chemical composition of organic extracts was accomplished using liquid chromatography, thin layer chromatography, and gas chromatography analysis. The in vitro test of three aqueous extract doses of *R. raphanistrum* against some apple and peach decline agents showed its efficacy in reducing mycelia growth. The in vivo assay of fine powder of this plant on peach seedlings revealed that treatment 8-weeks before the inoculation and planting was more efficient than the treatment before one week. This experiment revealed that the root weight of peach seedlings inoculated by *F. oxysporum* was improved to 207.29%. For apple seedlings, the treatment 8 weeks before the inoculation and plantation was more efficient than the treatment one week before; it reduced the root browning index. The study of *R. raphanistrum* chemical composition and its efficiency showed that the glucosinolates products: nitrile (4-Hydroxy-3-(4-methylphenylthio) butane nitrile, benzene acetonitrile, 4-fluoro,butane nitrile, 4-hydroxy-3-[(4-methylphenyl) thio] nitrile), and thiocyanate molecules (thiocyanic acid, ethyle) are responsible for the anti-fungal activities.

## 1. Introduction

Apple (*Malus domestica*) and peach (*Prunus persica*) crops occupy an important place in the world, in terms of total fruit yield within the fruit industry [1]. However, the development of these crops has had various phytosanitary problems (e.g., seedlings decline) [2,3]. In Tunisia, this problem was reported on apple trees in 2004, and the damage has since increased [4]. The damage observed in Tunisian apple orchards is caused by three species of *Phytophthora* (*Phytophthora cactorum*, *P. parasitica,* and *P. inundata*) and five *Pythium* species (*Pythium rostratifingens*, *P. irregulare*, *P. undulatum*, *P. indigoferae,* and *P. sterilum*) [4,5,6]. In Tunisian nurseries, the prevalence of *Fusarium*, *Pythium,* and *Phytopythium* species was associated with peach and apple seedling decline, demonstrating that these species were pathogens [7,8,9]. The protection of fruit tree plants against these pathogens could be managed by the application of fungicides [10,11,12]. However, these chemicals could lead to environmental pollution, the development of resistant strains, residual toxicity, and pathogen pressure [13]. As an alternative, biological control is the safest and economical method of plant pathogen management. This prompted intensive research on the development of biofungicides based on medicinal plants, as well as the antifungal activities of select plants. The therapeutic efficacy of plants is due to their organic compound metabolites for curing many diseases [14]. In fact, medicinal plants are characterized by the synthesis of substances that are useful for controlling microorganism development and promoting vegetable growth, and are a possible source of antimicrobial agents—non-phytotoxic, systemic, and biodegradable [15]. One promising way to achieve this goal is by using new tools based on plant bio-extracts to combat fungal diseases [14,15]. The use of allelochemical plant products in agricultural and horticultural practices can reduce the use of synthetic fungicides, associated potential for environmental contamination, and contribute to a more sustainable agricultural system [16]. In addition, the soil amendment by *Brassicaceae* seed meal provides systemic protection of apple seedlings against some elements of the fungal complex that cause the decline disease [3,16]. These results suggest that the incorporation and incubation of *Brassicaceae* plant residues in soils may be an appropriate means of controlling the elements of the microbial complex that causes apple and peach tree dieback disease in nurseries.

The aims of this study were to (i) evaluate the in vitro and in vivo efficacy of a *Brassicaceae* plant “*Raphanus raphanistrum*” against five pathogen species associated with apple and peach seedling decline in Tunisian nurseries; (ii) study the chemical composition of this plant; and (iii) determine the glucosinolates products responsible for this effect.

## 2. Results

### 2.1. In Vitro Evaluation of Aqueous Extract Against Fusarium and Oomycete Species

The mean diameter of *Pythium ultiumum* (*Py. ultimum*) and *Phytopythium mercuriale* (*Ph. mercuriale*) colonies, formed after two days and *Fusarium oxysporum*, *F. solani*, and *Phytophthora citrophthora* (*P. citrophthora*) colonies, measured after six days of incubation at 25 °C, varied according to aqueous extract doses tested and pathogen isolates, and are presented in Table 1. A highly significant interaction was observed between both fixed factors (pathogen isolates and extract doses) at *p* ≤ 0.001. In fact, the three aqueous extract doses reduced the mycelia growth of the apple and peach decline agents in comparison to their relative untreated controls. For the concentration of 5%, the growth reduction percent ranged between 55.19% for *F. solani* and 100% in case of *F. oxysporum*. For the 10% concentration, the growth reduction percent varied between 74.50% (*F. solani*) and 100% (*Py. ultimum*, *P. citrophthora*, and *F. oxysporum*). For the 15%concentration, the mycelial growth reduced by 77.88% in case of *P. mercuriale* and 100% for the other pathogens.

### 2.2. Effect of Raphanus raphanistrum Plant Extracts on Peach Seedling Decline Severity

The results of the effect of *R. raphanistrum* plant extracts on peach seedling decline severity revealed a highly significant effect of the pathogen species and the treatment date and significant (*p* ≤ 0.05) interaction (date of treatment×pathogens species) on the root browning index noted three months after the inoculation. However, the two treatment dates did not reduce this index for seedlings inoculated by the different (used) pathogens compared to the inoculated control.

The root weight registered three months after the inoculation of peach seedlings showed a significant effect on the pathogen species and a highly significant (*p* ≤ 0.001) effect on the treatment date without interaction (date of treatment * pathogen species). In fact, the percentage improvement of this parameter varied from 35.76% (recorded for the peach seedlings treated one week before their inoculation by *F. oxysporum*) to 207.29% (in the case of peach seedlings treated eight weeks before their inoculation by the same pathogen). Concerning the sanitary state index and the plant height, there were no significant effects for the two fixed factors or their interactions (Table 2).

### 2.3. Effect of Raphanus raphanistrum Plant Extracts on Apple Seedling Decline Severity

Three months post-inoculation, a highly significant effect of the two fixed factors (date of treatment and pathogen species) was noted. In the same sense, a highly significant (*p* ≤ 0.001) interaction (date of treatment × pathogen species) was obtained for apple seedling decline. The treatment 8 weeks before the inoculation and plantation was more efficient than the treatment before one week; it reduced the root browning index by 87.64% and 62.55% compared to the control inoculated by *Py. ultimum* and *Ph. mercuriale,* respectively. However, for the two treatment dates, neither the sanitary state nor the growth parameters of seedling inoculated by *Py. ultimum* or *Ph. mercuriale* improved (Table 3).

### 2.4. Chemical Composition of Raphanus raphanistrum

#### 2.4.1. Effect of *R. raphanistrum* Organic Extracts on Mycelial Growth of Pathogens Associated with Apple and Peach Decline in Tunisian Nurseries

**Figure 1:**
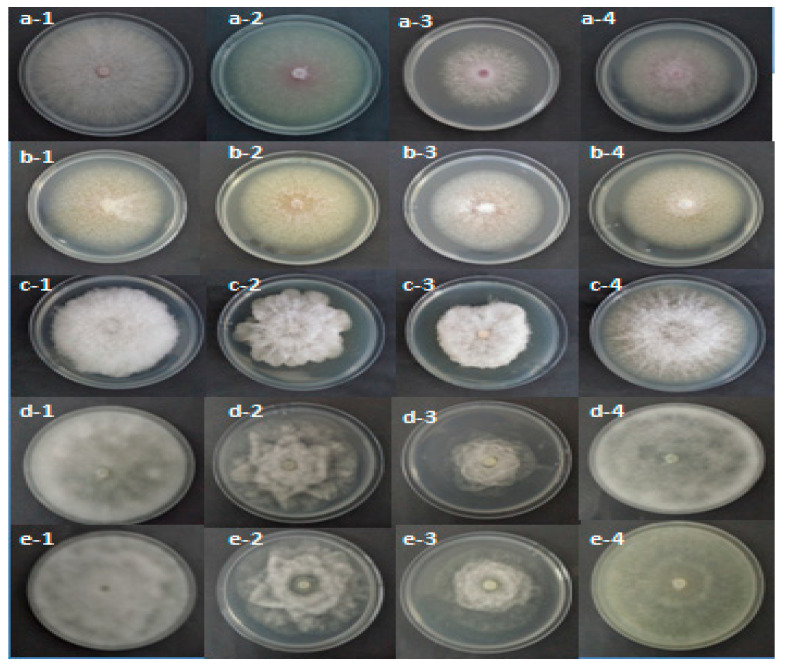
Effect of methanolic (2), dichloromethane (3) ethylacetate extracts (4) on mycelial growth of *F. oxysporum* (**a**), *F. solani* (**b**) *P. citrophthora* (**c**) recorded after six days of incubation and that of *Py. ultimum* (**d**) and *Ph. mercuriale* (**e**) noted after two days of incubation at 25 °C in comparison with the control (1).

Table 4 shows that, after two or six days, the pathogen mycelial growth was relatively affected by the *R. raphanistrum* organic extracts. Statistical analyses revealed highly significant effects of the organic extracts, the used pathogen species, and the interaction between these two fixed factors (*p* ≤ 0.001). In fact, the dichloromethane extract induced the lowest mycelial growth for all tested pathogens. The inhibition percent of mycelial growth was 75.29% for *Py. ultimum*, 27.3% for *F. solani*, 17.78% for *Ph. mercuriale,* and 67.58% for *P. citrophthora* (Table 4 and Figure 1).

#### 2.4.2. Liquid Chromatography (LC) and Thin Layer Chromatography Analysis

Ethyl acetate extract as well as the dichloromethane extract were fractionated on two columns of silica gel (LC) in 23 and 18 fractions, respectively. Their analysis by thin layer chromatography revealed that their compositions are less complex than those of the starting extract. The second simplification of the fractions chosen by their antifungal effect carried out to yield solid compounds white as lamellae and liquid compounds. The final number of fractions prepared for gas chromatographic (GC) analysis is 7.

#### 2.4.3. Effect of *Raphanus raphanistrum* Ethyl Acetate Extract Fractions on Mycelial Growth of Pathogens Associated with Apple and Peach Seedlings Decline in Nurseries

**Figure 2:**
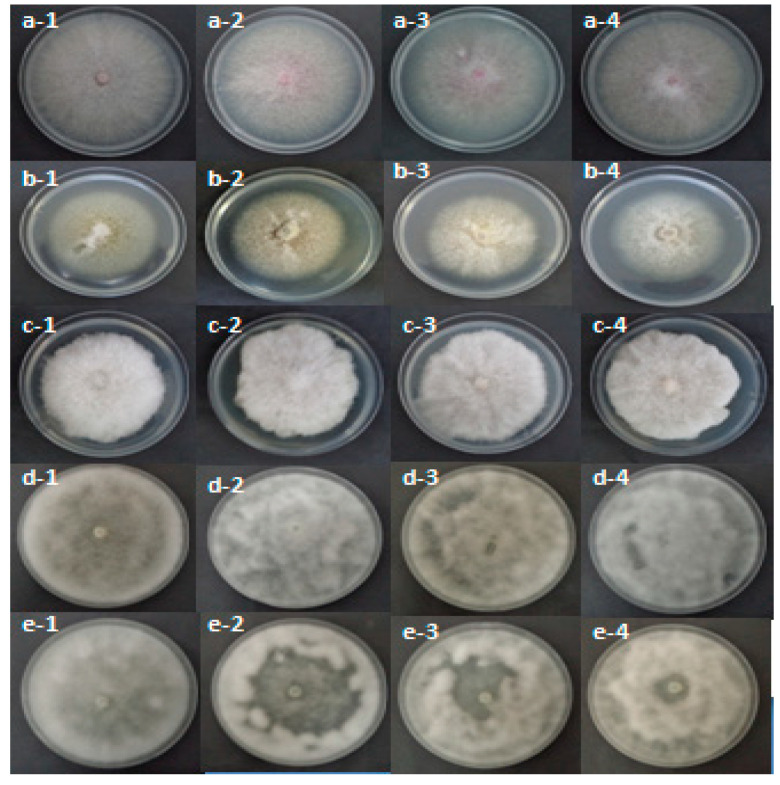
Effect of F1 (2), F12 (3) and F23 (4) fractions of *Raphanus raphanistrum* ethyl acetate extract on mycelial growth of *F. oxysporum* (**a**), *F. solani* (**b**) *P. citrophthora* (**c**) recorded after six days of incubation and that of *Py.ultimum* (**d**) and *Ph.mercuriale* (**e**) noted after two days of incubation at 25 °C in comparison with the control (1).

The test of the effect of 23 fractions of *R. raphanistrum* ethyl acetate extract against mycelial growth of the pathogens associated with apple and peach decline seedling nurseries showed that all fractions were inefficient. There is no inhibition for all treatments and pathogens. Figure 2 showed the effect of some fractions on mycelial growth of the studied pathogens. The absence of inhibitory effect prompted us to fractionate the dichloromethane extract to repair the fractions containing the antifungal effect of this plant.

#### 2.4.4. Effect of Raphanus raphanistrum Dichloromethane Extract Fractions on Mycelial Growth of Pathogens Associated with Apple and Peach Seedlings Decline in Nurseries

Mean diameter of pathogens colonies, developed after two and six days of incubation at 25 °C, seemed to be highly significantly varied according to dichloromethane extract fractions and pathogen isolates; a highly significant (*p* ≤ 0.001) interaction was also observed between both fixed factors at *p* ≤ 0.05. In fact, the test of 18 fractions of *R. raphanistrum* dichloromethane extract showed significant differences between the different treatments against all pathogens with the exception of *F. oxysporum*. In the case of *F. solani*, 11 fractions were effective with inhibition rates between 7.76% for F18 and 22.07% for F2. For *P. citrophthora*, ten fractions gave significant inhibition ranging from 22.94% for F16 to 66.38% for F11. For *Py. ultimum*, fourteen fractions significantly inhibited growth compared to the control. In fact, the most efficient fraction was F4 with an inhibition percent of 56.05%. While, inhibition of mycelial growth of *Ph. mercuriale* was exhibited by the 6 fractions with a rate varying between 20.28% (F11) and 31.11% (F9). Thus, the fractions that have an inhibitory effect on the majority of tested pathogens are F7, F8, F9, F10, F11, F12, F17 and F18 (Table 5).

#### 2.4.5. Gas Chromatography Analysis (GCA)

**Figure 3:**
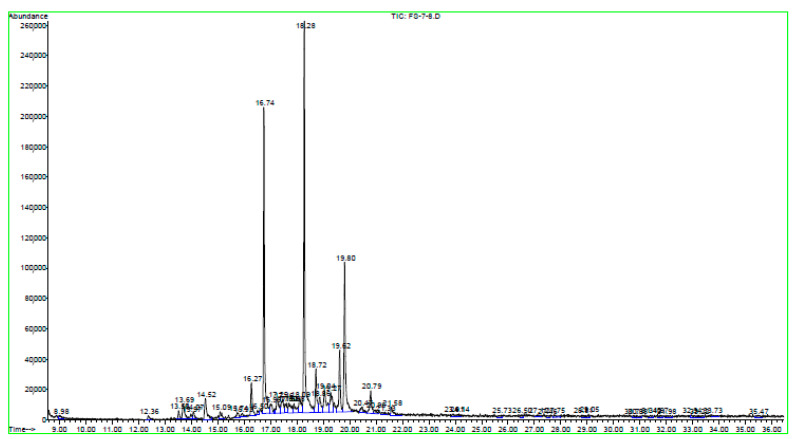
Chromatogram GCA of F7–8 dichloromethane extract fraction of *Raphanus raphanistrum* recorded on an apolar HP-5 column.

The chemical composition of the *R. raphanistrum* dichloromethane extract fractions was studied using GC. The Figure 3 represents the GCA chromatogram of the F7-8 fraction. The GCA chromatograms of the other fractions are shown in Supplementary Materials (Figures S1–S6). All the constituents highlighted were classified by types of organic compounds and their proportions were determined for each dichloromethane extract fraction of *R. raphanistrum* (Table 6). In this GC/MS study of the chemical composition of *R. raphanistrum* dichloromethane extract, many compounds were identified. The simplified extract into 7 fractions, represents a difference in the constituents and their percentages. The analysis of their antifungal efficacy on the five pathogens associated with apple and peach seedlings decline in Tunisian nurseries demonstrated an interesting result (Table 7).

**Figure 4:**
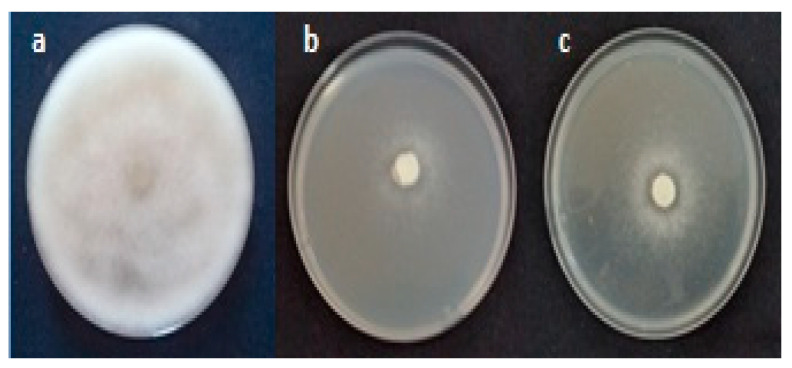
Inhibition of mycelial growth of *Pythium ultimum* in the presence of purified fractions F11 (**b**) and F17 (**c**) of *Raphanus raphanistrum* dichloromethane extract recorded after 3 days of incubation at 25 °C compared to the control (**a**).

The three oomycetes species were more susceptible to dichloromethane fractions than *Fusarium* species. The four first fractions (F7–8; F9; F10 and F11) were the most effective. It decreased the mycelial growth of the three *Pythiaceae* species. Nevertheless, the other fractions (F12; F17 and F18) were efficient only against two pathogens (Table 7 and Figure 4).

Two glucosinolate derivatives (nitrile and thiocyanate) were found in the most efficient fractions. In fact, the nitrile compounds exist in the fractions F7–8, F10, and F11. Thiocyanates exist only in the fraction F9 (Table 6 and Table 8). The fraction F9 is the most efficient. It reduced the mycelium growth of *P. citrophthora, Ph. mercuriale,* and *Py. ultimum* by 51.38; 51.74 and 31.11% respectively (Table 7). So, the glucosinolates products: nitrile (4-Hydroxy-3-(4-methylphenylthio) butanenitrile, Benzeneacetonitrile, 4-fluoro, Butanenitrile, 4-hydroxy-3-[(4-methylphenyl) thio] nitrile) and thiocyanate molecule (Thiocyanic acid, ethyle) are responsible of the antifungal activities of *Raphanus raphanistrum*.

## 3. Discussion

The three doses of *R. raphanistrum* aqueous extract were able to inhibit the in vitromycelial growth of *F. oxysporum*, *F. solani*, *Phytophthora citrophthora*, *Pythium ultimum*, and *Phytopythium mercuriale*. Moreover, results proved that the treatment of the substrate composed of 50% of soil, 25% of peat, and 25% of sand, 8 weeks before inoculation and planting of apple seedlings, was more effective than the treatment before one week. It reduced the root browning index compared to the inoculated control. These results are in agreement with previous studies demonstrating that soil treatment with *Brassicaceae* seed meal and duration of the incubation period prior to pathogen introduction significantly influenced apple seedling root infection. In fact, apple seedlings cultivated in soil infested with *P. abappressorium* 2–8 weeks after *Brassica juncea* seed meal amendment exhibited a significantly lower incidence of root infection by the introduced pathogen relative to the non-treated control [3,16,17,18,19].

In addition, the influence of *Brassicaceae* seed meal particle size on pathogen suppression has been reported but it varies depending on the mechanism of action [17]. Mazzola and Zhao [20] demonstrated that the *Pythium* spp. was well controlled in soil treated with fine particle sized seed meal. Additionally, they revealed that the particle size influenced the suppression of *R. solani* AG-5 but had no effect on the biological mechanisms of this pathogen suppression. A part of this finding corresponded with the result obtained in the current study with the use of fine particle sized *R. raphanistrum* plants. The test of this plant in vivo, against the peach seedling decline severity revealed an improvement of the root weight noted three months after the inoculation. Mazzola and Mullinix [21] proved that 3-year *Brassica napus* green manure significantly enhanced vegetative growth and yield of apple plants. However, the resulting disease control and growth response were inferior to that achieved by pre-plant methyl bromide soil fumigation. These results suggest that the incorporation and the incubation of residues of this plant in soils could be an appropriate tool of controlling microbial complex elements associated with apple and peach nursery decline. The study of the chemical composition of *R. raphanistrum* and its efficiency showed that themost efficient fractions contain nitrile and thiocyanate. These two products are produced by the hydrolysis of glucosinolates.

Previous studies revealed that all species of the *Brassicaceae* family are able to produce glucosinolates, which are generally considered as responsible for pathogen control because during their hydrolysis, they produce biologically active products including isothiocyanate, thiocyanate and nitrile [22,23,24].

Moreover, several studies have demonstrated the inhibition of plant pathogenic fungal agents during exposure to volatile substances produced by glucosinolate degradation at plant residues [25,26,27,28,29]. Besides, the inhibition of *R. solani* and *Pythium* spp., two elements of the fungal complex that cause apple decline disease, was achieved by exposure to volatile glucosinolate derivatives [28,29].

## 4. Materials and Methods

### 4.1. Used Pathogens

Two species of *Fusarium* (*F. oxysporum*, *F. solani*), one species of *Pythium* (*Py. ultimum*), one species of *Phytopythium* (*Ph. mercuriale*), and one species of *Phytophthora* (*P. citrophthora*) were used in this investigation. These isolates were obtained from peach and apple seedling nurseries, showing declined symptoms, in Tunisia, and proved to be causative agents of this disease (Table 9).

### 4.2. Preparation of Raphanus raphanistrum Extracts

**Figure 5:**
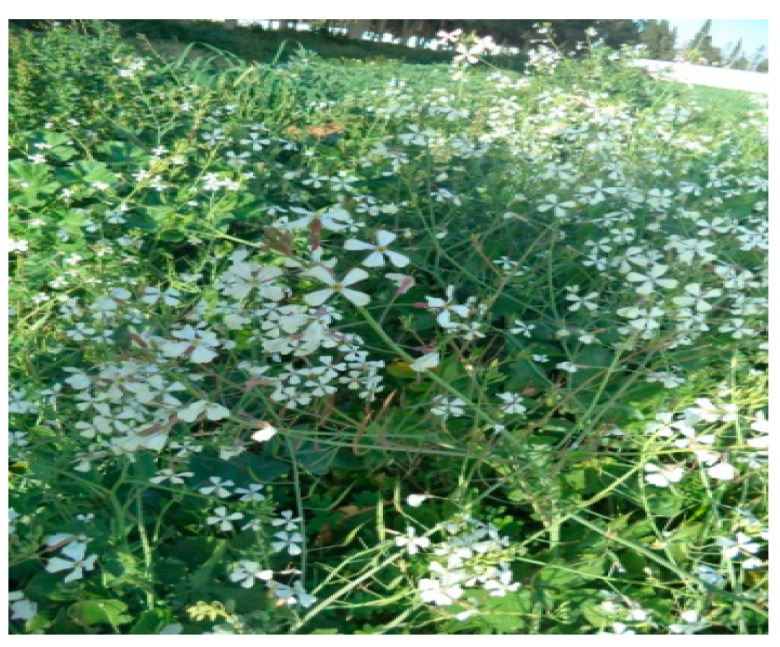
General aspect of *Raphanus raphanistrum*.

The studied plant (*Raphanus raphanistrum*) was harvested in March 2016 from the Chott-Mariem region (Sousse) (Figure 5). The plant, freshly harvested, was washed and dried in the shade in a dry and ventilated place for one month. Subsequently, the different parts of the plant were ground together with a fine mesh electric grinder to obtain a powder that was used for control trials. An aqueous extract was prepared by maceration in sterile distilled water. Thus, 50 g of powder was placed in a glass jar and macerated in 200 mL of sterile distilled water for 24 h at room temperature. The aqueous phase of the macerate was then filtered through a sterile micro filter to prevent contamination (Sigma-Aldrich, Merck KGaA, Darmstadt, Germany).

### 4.3. Effect of Raphanus raphanistrum Aqueous Extract on the Mycelial Growth of Agents Associated with Apple and Peach Seedlings Decline

The methodology of Mazzola and Zhao [19], with some modifications, was used to study the effect of *R. raphanistrum* aqueous extract on mycelial growth of different pathogens. The extracts were put into Erlenmeyer flasks containing potato dextrose agar (PDA) medium (45 °C) at different concentrations (5, 10, and 15%; *v*/*v*) and then the mixture was distributed into Petri dishes. For the control, the volume of the aqueous extract was replaced by the same volume of sterile distilled water. Then, agar plugs (6 mm in diameter) cut from 7-day-oldcultures of each pathogen were placed in the center of each plate. Three plates were performed for each concentration and for each isolate; the entire experiment was repeated twice. The culture plates were incubated at 25 °C for 3 days for *P. ultimum* and *P. mercuriale* and 6 days for *F. oxysporum*, *F. solani*, and *P. citrophthora*. The diameters of the colonies were measured and the percentage growth inhibition (PI) relative to the control was calculated according to the following formula:

PI = (1 − T/C) × 100(1)

where T: average diameter of the colonies in the presence of the plant extract, C: average diameter of the control colonies.

### 4.4. Effect of Raphanus raphanistrum Powder on Apple and Peach Seedling Decline Severity

To test the antifungal effect of this plant in vivo, the methodology of Mazzola and Zhao [21] was followed. This method consists to treat the substrate (50% sterilized soil, 25% sterilized peat and 25% sand) by 1% of *R. raphanistrum* fine powder and incubate it for 24 h in a closed plastic bag. Then, the substrate was divided into two parts. Each part was put in open bags at room temperature. The first one was incubated for 1 week while the second was incubated for 8 weeks. Four-week-old apple and peach seedlings, ‘MM106 and Garnem’, respectively, were transplanted into the treated substrate. These seedlings were grown in pots (23 cm diameter × 23 cm deep) in a greenhouse. To prepare the inoculums, ten discs of each pathogen of 7-day-old cultures grown on PDA medium, were incubated for one week in an Erlenmeyer flask containing 200 mL of potato dextrose broth (PDB), with stirring (120 rpm) (Dragon Laboratory, Corston, Bath, UK). The resulting suspensions were adjusted using a Molasses hemocytometer (Provision Labs’ Solutions, Charguia, Tunis, Tunisia). A volume of 50 mL of each pathogen inoculum adjusted to 10^6^ spores/mL for *Fusarium* isolates and 10^6^ zoospores/ml for *Pythiaceae* isolates was added to each plant. The experiment was conducted as a completely randomized design, and each elementary treatment was tested using three apple or peach seedlings.

After 90 days, apple and peach plants were uprooted, washed to eliminate the adhering peat and perlite, and examined. The disease severity was noted, according to the vegetative part sanitary state index and root browning index of plants. The sanitary state index rated on a 0–5 scale (0 = healthy seedlings, 1 = moderate discoloration of plant leaves (≤25%), 2 = moderate discoloration and falling leaves (≤50%), 3 = moderate discoloration of plant collar, stem, and leaves (≤75%), 4 = extensive discoloration of plant collar and stem with falling leaves (>75%), and 5 = dead plant) [30].However, the root browning index was rated according to a 0–5 scale (0 = no obvious symptoms, 1 = moderate discoloration of root tissue, 2 = moderate discoloration of tissue with some lesion, 3 = extensive discoloration of tissue, 4 = extensive discoloration of tissue with girdling lesions, and 5 = dead plant) [31]. For each seedling, the height and root weight were noted. Pathogen re-isolations using PDA and PARPBH medium were made from roots of inoculated plants to confirm Koch postulate.

### 4.5. Chemical Composition of Raphanus raphanistrum

#### 4.5.1. Preparation of *Raphanus raphanistrum* Organic Extracts

A total of 550 g of *R. raphanistrum* powder was packed in filter paper at a rate of 50 g per package and extracted with methanol by maceration in 2000 mL of cold methanol for ten days. The extract was concentrated in a rotary evaporator (Heidolph Instruments, Walpersdorfer, Schwabach, Germany) at 55 °C. Then, this extract was dried, weighed, and stored at 4 °C, protected from light until use.

Then, a volume of 100 mL of sterile distilled water and 50 mL of dichloromethane were added to the methanolic extract, and the mixture was placed in a separatory funnel. After1 h, the separation of the two phases was carried out, the lower phase containing dichloromethane, was recovered. A volume of 50 mL of dichloromethane was added to the separating funnel containing the remainder of the extract with water. After the separation of the phases, the dichloromethane phase was collected in the same Erlenmeyer flask to obtain a volume of 100 mL of dichloromethane containing the apolar molecules of *R. raphanistrum*. To extract the polar molecules, 50 mL of ethyl acetate was added to the ampoule containing water. This step was repeated twice. In each case, the upper phase containing the ethyl acetate extract was recovered. The extracts were concentrated in a rotary evaporator at 45 °C for dichloromethane and 60 °C for ethyl acetate. After the concentration, these extracts were dried for a few days at room temperature, then weighed and stored at 4 °C. The three organic extracts were tested in vitro, against pathogens species associated to apple and peach seedling decline in Tunisian nurseries. To evaluate the effect of these extracts, 10 mL of methanol were added to each dried extract. Then, the extracts were put in Erlenmeyer flasks containing the PDA medium (45 °C) at a concentration of 1% (*v*/*v*). Next, the mixtures were elapsed into Petri dishes. For the controls, the volume of the extract was replaced by the same volume of methanol. Then, agar plugs (6 mm in diameter) cut from 7-day-old cultures of each pathogen were placed at the center of each plate. Three plates were performed for each extract and for each isolate and the whole experiment was repeated twice in time. The culture plates were incubated at 25 °C for 3 days for *Py. ultimum* and *Ph. mercuriale* and 6 days for *F. oxysporum*, *F. solani,* and *P. citrophthora*. The diameter of pathogen colony was measured.

#### 4.5.2. Liquid Chromatography (LC) and Thin Layer Chromatography Analysis

Ethyl acetate and dichloromethane extracts were fractionated on two columns of silica gel (LC) in 23 and 18 fractions, respectively. These fractions were analyzed by thin layer chromatography and in vitro tested, against pathogens. Then, a second simplification of the most efficient fractions chosen by their antifungal effects was carried out by successive columns of silica gel using a mixture of dichloromethane and acetone (8/2; *v*/*v*). For this goal, a volume of 10 mL of methanol was added to each dried fraction. Then, the same used protocol for the test of organic extracts was followed.

#### 4.5.3. Gas Chromatography Analysis (GCA)

The GCA is the usual technique for extracts analysis. It allows to separate the volatile compounds from complex mixtures and undertaken a quantitative analysis of the results from a reduced injection volume. For each of compounds, two retention indices—polar and apolar—can be obtained. They were calculated from the retention times of a standard range of alkanes or rarely linear methyl esters at constant temperature [32] or by programming temperature (retention index: IR) [33]. They were then compared with those of reference products. The retention index of a compound A is defined by [34]:

(2) $$\mathrm{IR}=100N+100n\frac{\mathrm{Tr}\left(A\right)-\mathrm{Tr}\left(N\right)}{\mathrm{Tr}\left(N+n\right)-\mathrm{Tr}\left(N\right)}$$

where Tr(A): retention time of compound A, Tr(N) and Tr(N + n) are, respectively, the retention times of hydrocarbons of N carbons and N + n carbons such as: Tr(N) < Tr(A) < Tr(N + n).

The GC–MS analysis of seven fractions of dichloromethane extract was performed using a Hewlett-Packard GC–MS system (GC: 5890 series II; MSD 5972, Hewlett-Packard, Palo Alto, CA, USA) equipped with a HP-5 MS fused-silica capillary column (30 m × 0.25 mm, film thickness of 0.25 μm). Pure helium gas was used as a carrier gas at a constant flow rate of 1.2 mL/min. The oven temperature was programmed from 50 to 280 °C at 5 °C/min and, subsequently, held isothermal for 20 min. The injection port temperature was 250 °C and the detector was 280 °C.

Diluted samples (1/100, *v*/*v*, in dichloromethane) of 0.1 µL was injected in the split mode with a split ration 1:50. The relative percentage of the chemical constituents in the dichloromethane extract fractions was expressed as percentage by peak area normalization. Further confirmation was done from retention index data generated from a series of alkane retention indices (relative to C9–C28 on the HP-5 column, Sigma-Aldrich, Merck KGaA, Darmstadt, Germany).

### 4.6. Statistical Analysis

Statistical analyses (ANOVA) were performed for the in vitro and in vivo assays following a completely randomized factorial design where the pathogens species and the treatments (extract dose (test in vitro) or treatment date (test in vivo)) were the two fixed factors. Three replicates were used per individual treatment. For the study of *R. raphanistrum* chemical composition, the tests of the extracts and their fractions on pathogens mycelial growth were carried out according to a completely randomized design where the extracts or the fractions tested were the solo fixed factor and each individual treatment was replicated three times. Data analyses were performed using SPSS Software version 20 (IBM SPSS, Armonk, NY, USA)and mean separations were carried out using the S–N–K test (*p* < 0.05).Correlation analyses between the chemical composition of different *R. raphanistrum* fractions and the mycelial growth inhibition percentage in the presence of these fractions were carried out using Pearson’s correlation analysis at *p* ≤ 0.05.

## 5. Conclusions

The in vitro and in vivo evaluation of the effect of *R. raphanistrum* extracts against some apple and peach decline agents exhibited promising results. It improves the peach seedling root weight and reduces the root browning index of apple seedlings. The finding of our investigation demonstrated that the nitrile and thiocyanate molecules (derivatives of glucosinolates) are responsible for the anti-fungal activities.

## Figures and Tables

**Table 1:** Antifungal potential of three doses of *Raphanus raphanistrum* aqueous extract toward *P. ultimum* and *P. mercuriale* after 2 days and *F. oxysporum*, *F. solani* and *P. citrophthora* (Percent hyphal growth inhibition), noted after 6 days of incubation at 25 °C.

Concentration (%)	*Py. ultimum*	*Ph. mercuriale*	*P. citrophthora*	*F. solani*	*F. oxysporum*	*p*-Value ***
**5**	76.42 ± 1.09a * C **	71.15 ± 1.57aB	55.68 ± 1.25aA	55.19 ± 1.26aA	100.00 ± 0.00aD	≤0.001
**10**	100.00 ± 0.00bB	75.96 ± 1.11bA	100.00 ± 0.00bB	74.50 ± 3.34bA	100.00 ± 0.00aB	≤0.001
**15**	100.00 ± 0.00bB	77.88 ± 2.48bA	100.00 ± 0.00bB	100.00 ± 0.00cB	100.00 ± 0.00aB	≤0.001
***p*-value**	≤0.001	≤0.001	≤0.001	≤0.001	nd	

(*) Means ± standard error in the column followed by the same lower case letter (a, b) are not significantly different according to SNK test at *p* ≤ 0.05. (**) Means ± standard error in a row followed by the same capital letter (A, B, C, D) are not significantly different according to SNK test at *p* ≤ 0.05. (***) Probabilities associated with individual F tests. *p* -value listed in the last column refer to all five fungal species for the concentration in that row. *p*-value listed in the last row refer to all three concentrations for the pathogen in that column. (nd) not determined.

**Table 2:** Effect of *Raphanus raphanistrum* fine powder on the severity of decline disease and seedlings growth three months after the inoculation on peach seedlings ‘Garnem’.

	Treatment	*F. solani*	*F. oxysporum*	*Py. ultimum*	*P. citrophthora*	*p*-Value ***
**Root Browning Index**	**1W**	2.33 ± 0.58a * A **	1.41 ± 0.00Aa	2.33 ± 0.58Aa	3.67 ± 0.58Bb	0.015
	**8W**	2.33 ± 0.58Aab	1.67 ± 0.58Aa	1.67 ± 0.58Aa	3.33 ± 0.58Bb	0.023
	**NIC**	1.33 ± 0.58a	2.00 ± 0.00a	2.00 ± 0.00a	2.00 ± 0.00a	nd
	**IC**	2.00 ± 0.58a	1.33 ± 0.43a	2.00 ± 0.00a	2.33 ± 0.58a	nd
***p*-value**		0.119	0.219	0.330	≤0.001	
**Sanitary State Index**	**1W**	3.00 ± 1.00Aa	2.01 ± 0.00Aa	3.00 ± 0.00Aa	2.33 ± 0.58Aa	0.160
	**8W**	1.67 ± 0.58Aa	1.67 ± 0.58Aa	2.33 ± 0.58Aa	2.00 ± 0.00Aa	0.363
	**NIC**	1.67 ± 0.58a	2.33 ± 0.58a	2.33 ± 0.58a	2.33 ± 0.58a	nd
	**IC**	2.33 ± 0.58a	2.67 ± 0.83a	1.67 ± 0.58a	2.33 ± 0.58a	nd
***p*** **-value**		0.139	0.163	0.067	0.576	
**Height** **(cm)**	**1W**	53.83 ± 0.29Aa	56.13 ± 9.00Aa	64.67 ± 9.07Aa	56.17 ± 5.51Aa	0.285
	**8W**	58.67 ± 20.0Aa	82.17 ± 8.01Aa	58.00 ± 17.00Aa	64.67 ± 2.52Aa	0.192
	**NIC**	62.83 ± 6.79a	62.83 ± 6.79a	62.83 ± 6.79a	62.83 ± 6.79a	nd
	**IC**	48.00 ± 4.58a	59.17 ± 13.83a	61.17 ± 4.75a	52.50 ± 7.26a	nd
***p*-value**		0.423	0.135	0.881	0.09	
**Root Weight (g)**	**1W**	3.98 ± 0.21Aa	3.91 ± 0.26abA	7.18 ± 1.93abA	6.63 ± 2.21abA	0.117
	**8W**	4.18 ± 1.56Aa	8.85 ± 1.57Bb	7.33 ± 1.33Bb	6.75 ± 0.33abB	0.014
	**NIC**	7.38 ± 2.09b	7.38 ± 2.09b	7.38 ± 2.09b	7.38 ± 2.09b	nd
	**IC**	2.61 ± 0.78a	2.88 ± 1.52a	3.71 ± 0.26a	3.25 ± 0.36a	nd
***p*-value**		0.015	0.010	0.055	0.018	

(*) Means ± standard error in the column for each parameter (root browning index, sanitary state index, height and root weight) followed by the same lower case letter (a, b) are not significantly different according to the SNK test at *p* ≤ 0.05. (**) Means ± standard error in a row followed by the same capital letter (A, B) are not significantly different according to SNK test at *p* ≤ 0.05. (***) Probabilities associated with individual F tests. (nd) not determined. *p*-value listed in the last column refer to all 4 fungal species for the treatment in that row. *p*-value listed in the last row for each parameter refer to all treatments (1W, 8W, NIC, and IC) for the pathogen in that column. (NIC) uninoculated control; IC: inoculated control; 1W: substrate treatment one week before planting and inoculation; 8W: substrate treatment eight week before planting and inoculation.

**Table 3:** Effect of *Raphanus raphanistrum* on the severity of pathogens associated to apple seedling decline on apple seedlings ‘MM106’ and seedlings growth three months after the inoculation.

	Treatment	*Py. ultimum*	*Ph. mercuriale*
**Root Browning Index**	**1W**	5.00 ± 0.00d *	1.67 ± 0.58a
	**8W**	0.33 ± 0.58a	1.00 ± 0.00a
	**NIC**	1.33 ± 0.58b	1.33 ± 0.58a
	**IC**	2.67 ± 0.58c	2.67 ± 0.58b
*p*-value **		≤0.001	0.017
**Sanitary State Index**	**1W**	5.00 ± 0.00b	2.33 ± 0.58b
	**8W**	1.00 ± 0.00a	1.00 ± 0.00a
	**NIC**	1.33 ± 0.58a	1.33 ± 0.58ab
	**IC**	1.67 ± 0.58a	1.33± 0.58ab
***p*-value**		≤0.001	0.052
**Height (cm)**	**1W**	12.00 ± 2.00a	52.33 ± 5.01a
	**8W**	63.43 ± 3.10b	54.50 ± 4.77a
	**NIC**	90.33 ± 11.02c	90.33 ± 11.02b
	**IC**	62.00 ± 6.00b	80.67 ± 2.08b
***p*-value**		≤0.001	≤0.001
**Root Weight (g)**	**1W**	0.34 ± 0.13a	4.87 ± 0.34a
	**8W**	5.53 ± 0.11b	4.36 ± 1.24a
	**NIC**	11.81 ± 1.32d	11.81 ± 1.32b
	**IC**	7.05 ± 0.06c	11.64 ± 0.45b
***p*** **-value**		≤0.001	≤0.001

(*) Means ± standard error in the column for each parameter (root browning index, sanitary state index, height and root weight) followed by the same lower case letter (a, b, c, d) are not significantly different according to SNK test at *p* ≤ 0.05. (**) Probabilities associated with individual F tests. (NIC) uninoculated control; IC: inoculated control; 1W: substrate treatment one week before planting and inoculation; 8W: substrate treatment eight week before planting and inoculation.

**Table 4:** Antifungal potential (percent hyphal growth inhibition) of *Raphanus raphanistrum* organic extracts toward *Py. ultimum* and *Ph. mercuriale* noted after 2 days and *F. oxysporum*, *F. solani*, and *P. citrophthora* noted after 6 days of incubation at 25 °C.

	*F. oxysporum*	*F. solani*	*P. citrophthora*	*Py. ultimum*	*Ph. mercuriale*	*p*-Value ***
**EM**	5.41 ± 1.32a * A **	6.90 ± 1.33aA	27.52 ± 7.16bC	41.57 ± 4.29bD	15.45 ± 2.43bB	≤0.001
**EA**	15.95 ± 2.35bC	18.68 ± 1.72bC	1.22 ± 1.04aA	29.36 ± 5.06aD	6.12 ± 2.23aB	≤0.001
**ED**	25.93 ± 1.32cB	27.30 ± 1.10cB	67.58 ± 5.14cC	75.29 ± 5.57cD	17.78 ± 2.02bA	≤0.001
***p*-value**	≤0.001	≤0.001	≤0.001	≤0.001	≤0.001	

(*) Means ± standard error in the column followed by the same lower case letter (a, b, c) are not significantly different according to SNK test at *p* ≤ 0.05. (**) Means ± standard error in a row followed by the same capital letter (A, B, C, D) are not significantly different according to SNK test at *p* ≤ 0.05. (***) Probabilities associated with individual F tests. *p*-value listed in the last column refer to all 5 fungal species for the extract in that row. *p*-value listed in the last row refer to all extracts for the pathogen in that column. EM: Methanolic extract, EA: Ethyl Acetate extract, ED: Dichloromethane extract.

**Table 5:** Effect of *Raphanus raphanistrum* dichloromethane extract fractions on mycelial growth (cm) of *Fusarium oxysporum*, *F. solani*, *P. citrophthora* recorded after six days of incubation and that of *Py.ultimum* and *Ph. mercuriale* noted after two days of incubation at 25 °C.

Fractions	*F. oxysporum*	*F. solani*	*P. citrophthora*	*Py. ultimum*	*Ph. mercuriale*	*p*-Value ***
**Control**	8.03a *	8.70a	8.18a	8.60a	9.00a	nd
**F1**	7.98aB **	7.28eA	8.13aB	7.58aA	9.00aC	≤0.001
**F2**	8.00aD	6.78fC	5.33c-fA	5.90bcdB	8.90aE	≤0.001
**F3**	7.95aB	8.63aC	8.08aB	6.55bA	9.00aD	≤0.001
**F4**	8.03aC	7.45deB	6.80abcB	3.78fA	7.00bB	≤0.001
**F5**	8.03aB	8.30abB	5.98bcdA	5.65bcdA	8.90aC	≤0.001
**F6**	7.88aB	7.73cdB	8.13aB	6.23bcA	8.88aC	≤0.001
**F7**	8.00aC	7.20eBC	8.08aC	5.25cdeA	6.38cAB	≤0.001
**F8**	7.78aC	7.40deC	4.15efA	3.90fA	6.25cB	≤0.001
**F9**	7.68aC	7.23eBC	3.98fA	4.15efA	6.20cB	≤0.001
**F10**	7.85aD	7.98bcD	5.55cdeB	4.43efA	6.45cC	≤0.001
**F11**	7.73aC	8.55aD	2.75gA	3.90fB	7.18bC	≤0.001
**F12**	7.93aD	7.48deC	3.95fA	4.83defB	8.90aE	≤0.001
**F13**	8.03aC	8.55aCD	7.18abB	6.40bcA	9.00aD	≤0.001
**F14**	8.03aB	8.65aB	4.55defA	8.65aB	9.00aB	≤0.001
**F15**	8.00aB	8.73aC	6.80abcA	8.28aB	8.98aC	≤0.001
**F16**	8.00aB	7.78cdB	6.30bcA	8.23aB	9.00aC	≤0.001
**F17**	7.95aB	8.50aB	5.75bcdA	6.28bcA	9.00aB	≤0.001
**F18**	7.88aB	8.03bcB	4.23efA	4.13efA	8.90aC	≤0.001
***p*-value**	=0.08	≤0.001	≤0.001	≤0.001	≤0.001	

(*) Means ± standard error in the column followed by the same lower case letter (a, b, c, d, e, f) are not significantly different according to SNK test at *p* ≤ 0.05. (**) Means ± standard error in a row followed by the same capital letter (A, B, C) are not significantly different according to SNK test at *p* ≤ 0.05. (***) Probabilities associated with individual F tests, nd: not determined. *p*-value listed in the last column refer to all 5 fungal species for the fraction in that row. *p*-value listed in the last row refer to all fractions for the pathogen in that column.

**Table 6:** Identified constituents classified by types of organic compounds and corresponding proportions.

Constituents (%)	F7–8	F9	F10	F11	F12	F17	F18
**Carboxylic acids**	0.00	0.69	0.00	0.00	0.00	11.81	0.00
**Alcohols**	5.73	7.63	21.79	10.98	8.19	9.61	6.49
**Aldehydes**	0.63	6.32	1.12	1.65	4	0.00	0.29
**Amines**	0.00	13.23	3.20	1.23	0.00	0.00	0.35
**ketones**	7.44	1.77	1.08	0.00	1.9	9.68	9.47
**Esters**	4.32	7.9	3.93	3.96	0.00	8.46	6.01
**Unsaturated hydrocarbons**	43.16	26.44	25	63.43	3.19	37.93	0.00
**Saturated hydrocarbons**	1.15	1.60	1.62	2.44	0.00	0.00	10.24
**Ethers**	0.00	0.00	0.00	0.00	8.21	1.27	0.33
**Dimethyl sulfoxide**	0.00	5.68	0.00	0.00	0.00	0.00	0.00
**Nitriles**	0.82	0.00	2.7	0.52	0.00	0.00	0.00
**Amides**	0.00	0.00	0.00	2.19	0.00	0.00	0.00
**Indoles**	0.00	11.55	0.00	0.00	3.81	0.00	0.00
**Thiocyanates**	0.00	3.93	0.00	0.00	0.00	0.00	0.00

**Table 7:** Mycelial growth inhibition percent of pathogens tested in the presence of purified *Raphanus raphanistrum* dichloromethane extract fractions.

Pathogens				Fractions			
	F7–8	F9	F10	F11	F12	F17	F18
***F. oxysporum***	1.56 ± 1.76 ^a^ *	4.36 ± 3.43 ^ab^	2.41 ± 2.66 ^a^	17.67 ± 2.34 ^d^	7.44 ± 2.34 ^b^	12.56 ± 2.15 ^c^	1.87 ± 1.87 ^a^
***F. solani***	14.94 ± 1.63 ^ab^	16.95 ± 5.26 ^b^	8.33 ± 0.57 ^a^	13.58 ± 8.55 ^ab^	12.35 ± 1.43 ^ab^	19.75 ± 1.43 ^b^	7.76 ± 1.45 ^a^
***P. citrophthora***	25.06 ± 3.56 ^a^	51.38 ± 15.99 ^b^	32.11 ± 5.04 ^a^	50.00 ± 0.76 ^b^	44.66 ± 5.20 ^b^	48.09 ± 2.79 ^b^	48.32 ± 7.23 ^b^
***Ph. mercuriale***	38.95 ± 3.65 ^c^	51.74 ± 8.08 ^d^	48.55 ± 3.34 ^d^	20.12 ± 0.68 ^b^	3.99 ± 3.29 ^a^	13.31 ± 7.57^b^	52.03 ± 2.58 ^d^
***Py. ultimum***	29.10 ± 1.14 ^b^	31.11 ± 3.27 ^b^	28.33 ± 4.93 ^b^	0.00 ± 0.00 ^a^	45.99 ± 0.00 ^c^	0.00 ± 0.00 ^a^	1.11 ± 1.57 ^a^

(*) Means ± standard error in a row, followed by the same lower case letter (a, b, c, d) are not significantly different according to SNK test at *p* ≤ 0.05.

**Table 8:** Glucosinolate degradation products in the fractions identified by Gas Chromatography Analysis (GCA).

Fractions	Family	Noun	Percentage	Structure
**F7–8**	Nitrile	4-Hydroxy-3-(4-methylphenylthio) butanenitrile	0.82	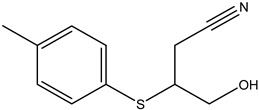
**F9**	Thiocyanate	Thiocyanic acid, ethyle	3.93	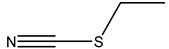
**F10**	Nitrile	Benzeneacetonitrile, 4-fluoro	2.7	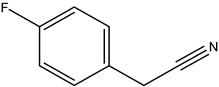
**F11**	Nitrile	Butanenitrile, 4-hydroxy-3-[(4-methylphenyl)thio]nitrile	0.52	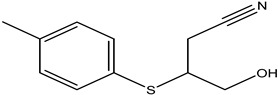

**Table 9:** Pathogen isolates collected from peach and apple seedling nurseries and used in this investigation.

Species	Isolates	Origins	Sampling Years	GenBank Accession Number
***F. oxysporum***	Fo22	peach	2013	MF993097
***F. solani***	F48	peach	2012	MF993094
***Pythium ultimum***	P42	peach	2013	MF993110
	Po2	apple	2012	MH260594
***Phytopythium mercuriale***	Po26	apple	2013	MF993112
***Phytophthora citrophthora***	P39	peach	2013	nd

nd: not determined.

## Data Availability

The data associated with the present manuscript are included in the Supplementary Materials.

## Supplementary Materials

The following are available online. Tables S1–S7: Constituents of the fraction F1–18 identified by Gas Chromatography Analysis (CPG). Figures S1–S6: Chromatogram CPG of F1–18 dichloromethane extract fraction of *R. raphanistrum* recorded on an apolar HP-5 column.

## Sample Availability

Samples of the compounds are not available from the authors.
