# Supplementary material for: Analysis of Chemical Composition and In Vitro and In Vivo Antifungal Activity of Raphanus raphanistrum Extracts against Fusarium and Pythiaceae, Affecting Apple and Peach Seedlings
Source: Molecules. 2021 Apr 23;26(9):2479. doi: 10.3390/molecules26092479 (PMC8123050; doi:10.3390/molecules26092479)
Supplement: Supplementary file 1 [file molecules-26-02479-s001.zip › molecules-1125591-supplementary.pdf]

Table S1. Constituents of the fraction F7–8 identified by Gas Chromatography Analysis (CPG).

Noun	Retention Time	percentage	structure
Alcohols			
Hexadecanol	21,58	1,54	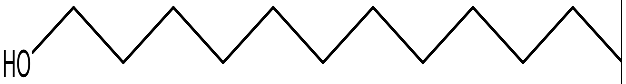
4-Hydroxy-3-(4-methylphenylthio) butanenitrile	33,22	0,82	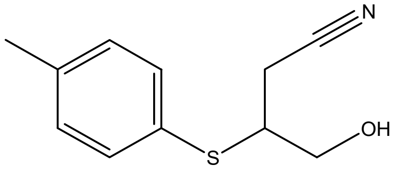
Pentadecanol	15,09	0,68	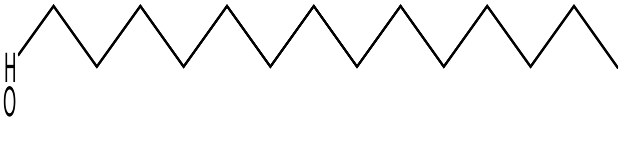
2,5-Di-tert-butylhydroxybenzene	16,27	2,69	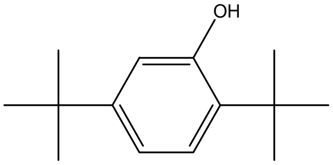
Aldehydes			
Citronellyl formate	14,07	0,63	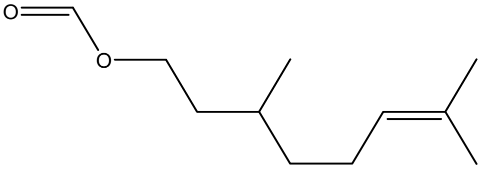
Cetones			
4-Hydroxy-2-methylacetophenone	14,52	2,34	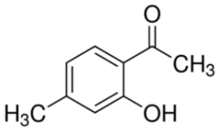
6,10-Dimethylundecan-2-one	18,72	3,52	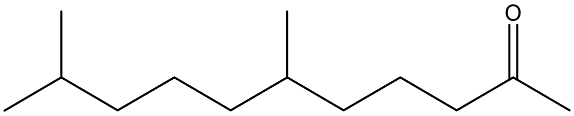
Ester			
Methyl oleate	20,79	2,05	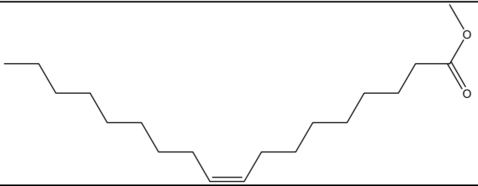
Methyl 9-oxononanoate	19,27	2,27	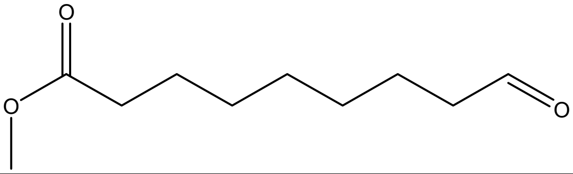
Ethers			

Hexadecene epoxide	18,86	1,58	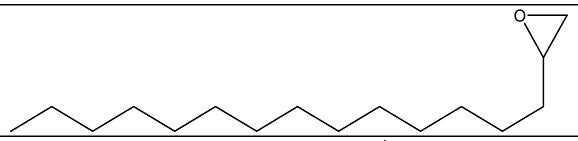
2-Isobutyl-3-methylfuran	17,60	1,18	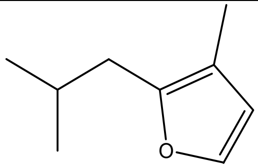
Unsaturated hydrocarbons			
4-Methyl-1,4-heptadiene	13,69	1,82	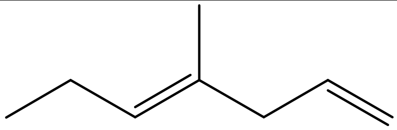
1-Octadecene	18,28	23,64	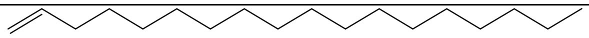
1-Hexadecene	16,74	17,71	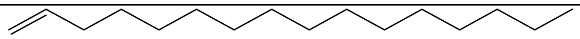
Saturated hydrocarbons			
Methylcyclooctane	16,99	1,15	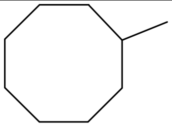

Table S2. Constituents of the fraction F9 identified by Gas Chromatography Analysis (CPG) .

Noun	Retention Time	Percentage	Structure
Carboxylic Acids			
Acide -2-méthylundec-10-énoique	15,59	0,69	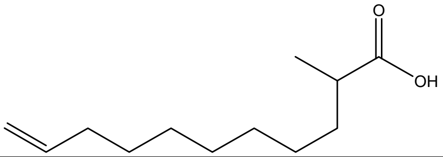
Alcohol			
Hexahydrofarnesol	17,80	1,77	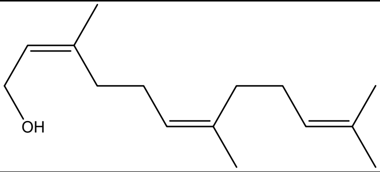
1-Octanol, 2-butyl	15,70	0,87	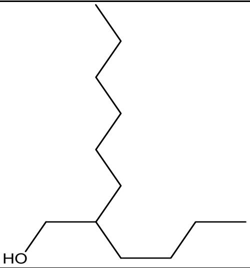
2,4-Di-tert-butylphenol	16,29	2,98	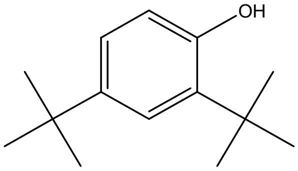
1-Octadecanol	13,25	0,76	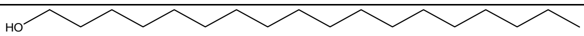
7-HYDROXYDISPIRO(2.0.2.2)OCTANE	13,45	0,74	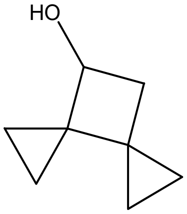
2-Hexyn-1-ol	12,00	0,51	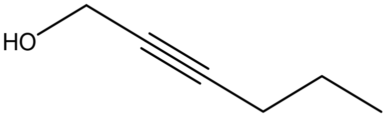
Aldehydes			
2-PENTYNAL	17,24	1,2	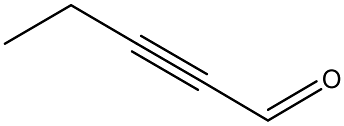
beta.-Methylcrotonaldehyde	13,37	0,53	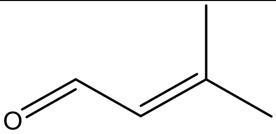
2-Decenal, (E)	14,06	4,59	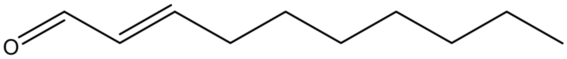
Amine			

Azetidine, 1,2-dimethyl	9,19	1,73	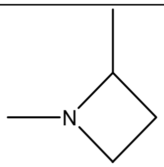
1H-Indole	14,71	11,55	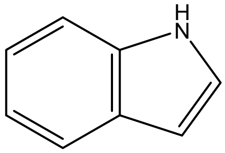
Cetones			
3-ETHYLCYCLOPENT-2-EN-1-ONE	14,36	0,80	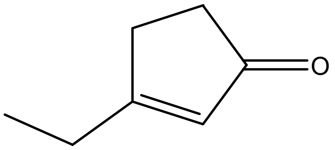
Hexahydrofarnesyl ac etone			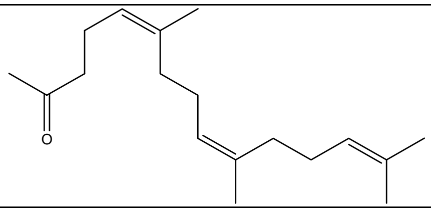
Ester			
Hexadecanoate de méthyle	19,29	3,97	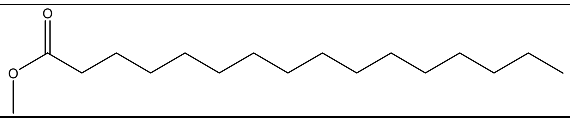
Ether			
Furan, 2-pentyl	11,23	1,57	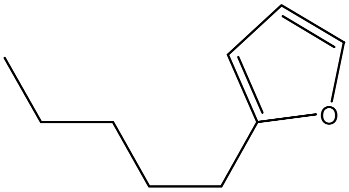
Unsaturated hydrocarbons			
4,4-Dimethyl-cis-2-pentene	15,88	1,88	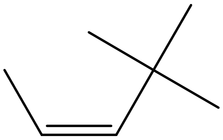
1-Hexadecene	16,76	8,61	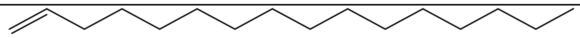
Ethylbutyl acetylene	16,98	2,59	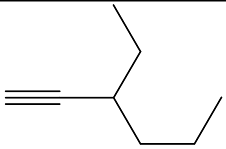
1,5-Heptadiene, 2-methyl-, (Z)	18,06	1,31	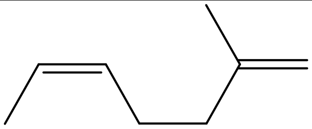
1-Octadecene	18,27	5,30	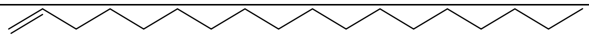
1-Tetradecene	15,10	3,59	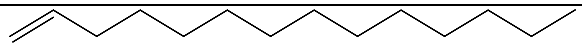

(4R,5S)-4-(1-propyn-1-yl)-5-vinylcyclohexene	15,34	2,39	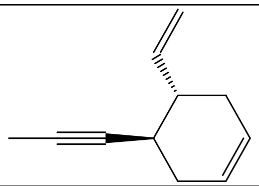
1,2-Pentadiene, 4,4-dimethyl	12,44	0,77	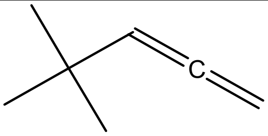
Saturated hydrocarbons			
Isobutylene dichloride	14,98	1,60	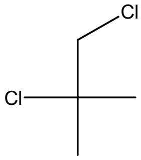
Others			
Methane, sulfinylbis	9,81	5,68	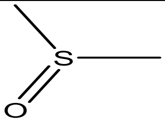
Thiocyanic acid, ethyle	13,76	3,93	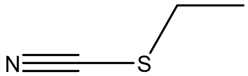
DIMETHYLPHOSPHINIC ACID<br>acide	10,63	0,65	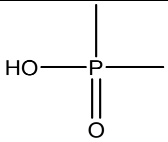

Table S3. Constituents of the fraction F10 identified by Gas Chromatography Analysis (CPG).

Noun	Retention Time	Percentage	Structure
		Alcohols	
1,3-Benzenediol, 3-methyl	18,72	16,70	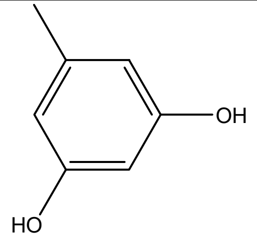
1,12-Dodecanediol	20,77	0 ,77	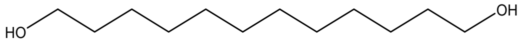
Phenol, 2,4-bis(1,1-dimethylethyl)	16,26	3,82	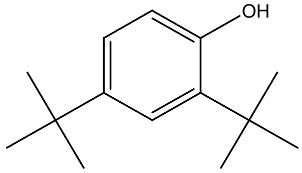
beta.-Citronellol	16,55	0,50	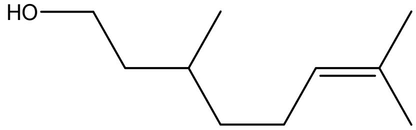
		Aldehyde	
3-(1-Nitro-2-oxocyclohexyl)propanal	13,71	1,12	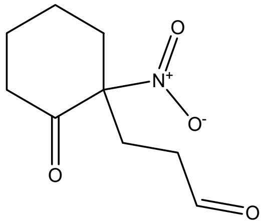
		Amine	
Benzenamine, 2-methyl	17,70	3,20	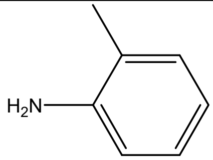
		Cetone	
1-Propanone, 2-methyl-1-pyrazinyl	17,22	1,08	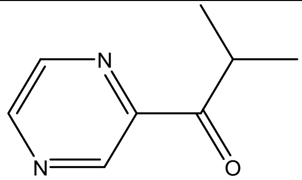
		Ester	
8-oxooctanoate de méthyle	19,27	0,88	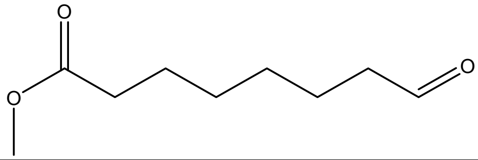
		Ethers	

Furan, 2-(methoxymethyl)	11,18	2,12	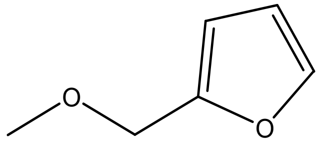
Tetrahydro-2-methyl-3-methylene-furan	14,02	1,81	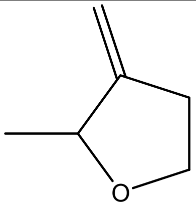
Unsaturated hydrocarbons			
1-Hexadecene	16,74	13,61	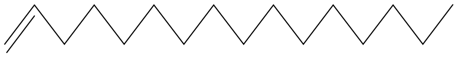
7-chloro Hept-3-yne	13,86	0,73	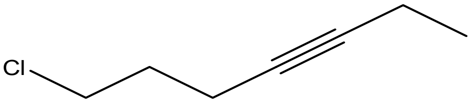
Pentatriacont-17-ene	21,57	0,81	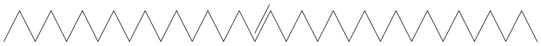
Octadecene	18,25	10,22	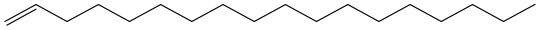
Saturated hydrocarbons			
Decane, 2-methyl	15,67	0,81	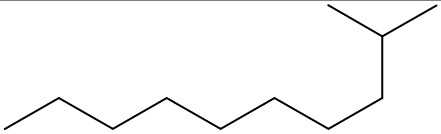
Hexadecane, 1-chloro	16,98	0,81	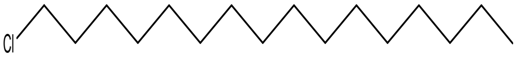
Nitrile			
Benzeneacetonitrile, 4-fluoro	14,53	2,70	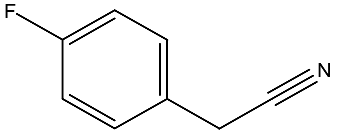

Table S4. Constituents of the fraction F11 identified by Gas Chromatography Analysis (CPG) .

**Noun**	**Retention Time**	**Percentage**	**Structure**
		Alcohols	
6-Methyl-1-heptanol	15,10	1,34	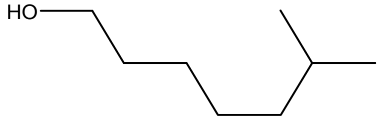
Phenol, 3,5-bis(1,1-dimethylethyl)	16,27	4,09	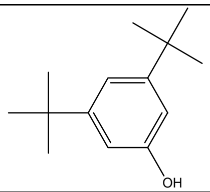
2-Heptyn-1-ol	17,26	0,82	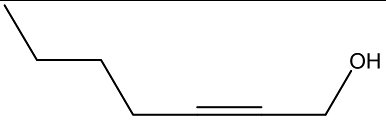
1,5-Pentanediol, 3-methyl	18,86	1 ,10	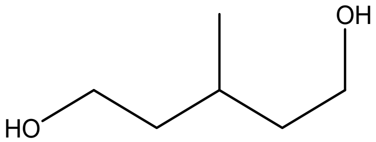
trans -2-Hepten-1-ol	20,78	0,99	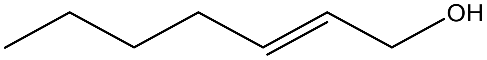
Hexacosylalcohol	21,57	1,44	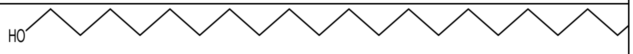
1-Hexadecanol	27,84	0,51	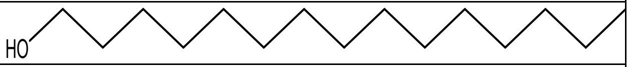
4-Nitro-2,6-dichlorophenol	32,75	0,69	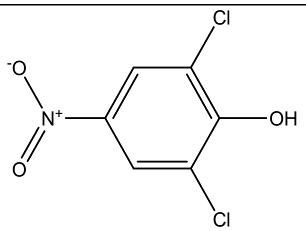
		Aldehyde	
2-trans-Hexenal	17,02	1,65	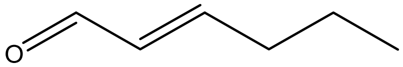
		Amides	
4-Aminobutyric acid lactam	10.24	0,94	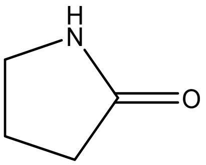
(1RS,2RS,3RS,7RS)-1,2,6,6-tetramethyl-10-oxatricyclo[5.2.1.0(2,7)]dec-3-yl N-phenylcarbamate	26,82	1,25	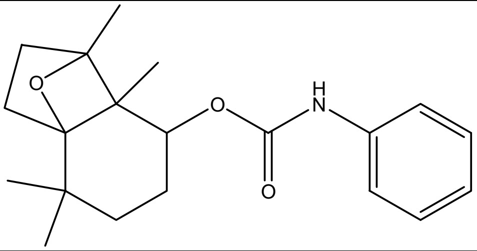
		Amines	

Azetidine, 1-bromo-<br>amine	32,23	0,75	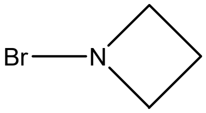
Isoxazolidine, 4-(fluoromethylene)-2-methyl-3-phenyl-, (Z)-<br>amine	33,38	0,48	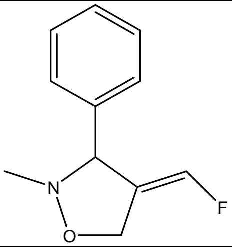
Esters			
methyl 2-bromo-2-butenoate	19,61	1,30	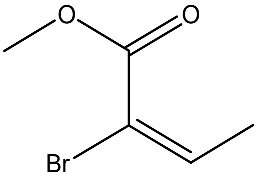
ETHYL 2-METHYL-3-OXOHEXANOATE	10,50	2,66	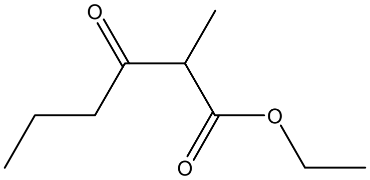
Unsaturated hydrocarbons			
1,cis-3-Pentadiene	16,54	0,66	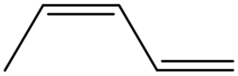
1-Hexadecene	16,74	29,13	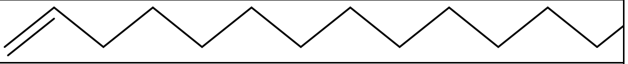
9-Octadecene, (E)	18,25	26,26	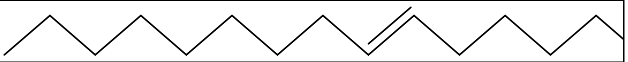
17-Pentatriacontene	19,79	7,37	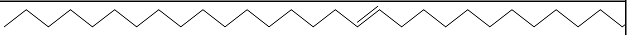
Saturated hydrocarbons			
Silane, trichloroeicosyl	17,40	0,55	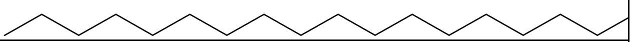
Decyl fluoride	19,34	1,37	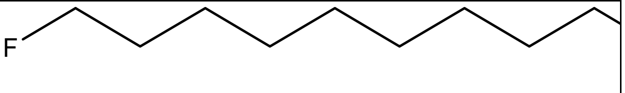
Nitrile			
Butanenitrile, 4-hydroxy-3-[(4-methylphenyl)thio]<br>nitrile	23,87	0,52	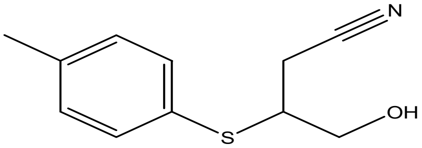

Table S5. Constituents of the fraction F12 identified by Gas Chromatography Analysis (CPG).

Noun	Retention Time	Percentage	Structure
Alcohols			
3-methoxyphenol	18.79	2.01	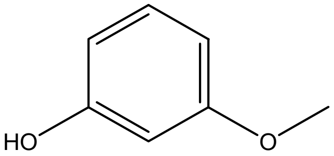
2,5-Di-tert-butylhydroxybenzene Phenol, 2,4-bis(1,1-dimethylethyl)	16.24	5.24	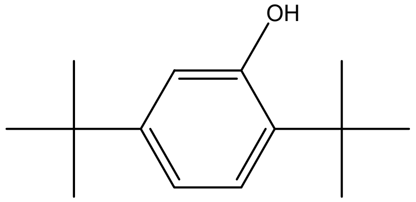
1-Tetracosanol	18.07	0.95	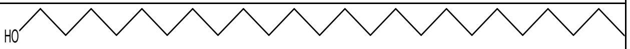
Aldehydes			
Tetradecanal	19.23	2.90	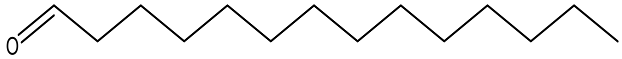
n-Dodecyl aldehyde	16.94	1.10	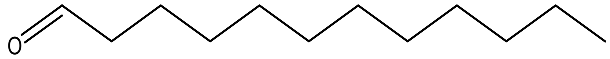
Cetone			
3,9,10-Tribromo-(+)-camphor cetone	21.53	1.90	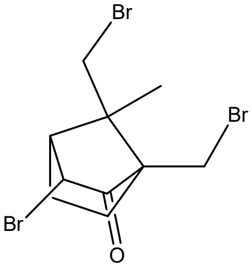
Ethers			
Spiro[7-oxabicyclo[4.1.0]heptane-2,2'-oxirane], 1,5,5-trimethyl-6-(3-methyl-1,3-butadienyl)-, [1.alpha.,2.alpha.,6.alpha.(E)]	17.90	0.99	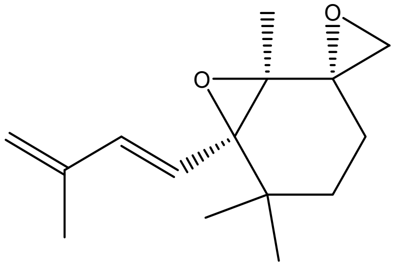
Epoxycycloheptane	20.74	2.58	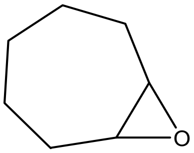
1-methoxy-2-methylidenecyclopropane	11.01	2.90	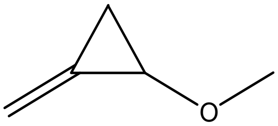

1,3-Dihydro-1-ethoxy-1-methoxyisobenzofuran	20.37	1.74	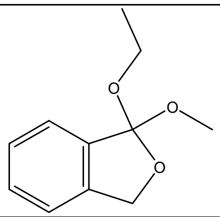
Unsaturated hydrocarbons			
4,4-Difluoro-4-iodo-2,3,3-trimethyl-1-butene	18.51	0.80	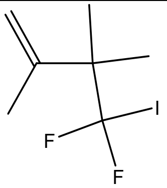
HEXADECENE-1	16.72	9.39	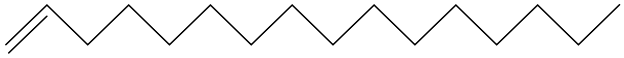
Saturated hydrocarbons			
Tetradecyl chloride hydrocarburesaturé	19.13	0.83	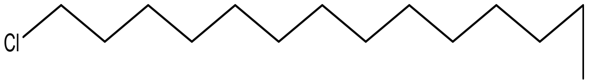
Others			
2-(2-dimethylaminoethyl)-3-cyanome thyl-5-methylindole indole	18.68	3.81	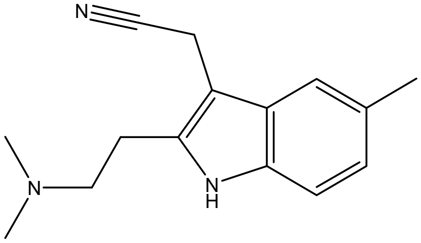
Dioctadecyl phosphonate	18.23	13.77	
1-methyl-2-carboxaldehyde-3(1-carboxaldehyde)-ethenyl-cyclopentane	17.73	1.39	
Oct-2-yn-1-o 2-Nitro-1-decen-4-yne	17.51	2.41	
Octanoic acid, 7-chloro-, chlorome thyl ester	17.21	1.48	
Furo[3,4 -c]pyridine, 1,3-dihydro-6-methyl-7(trimethylsilyl)-Azetidine, 2-methyl-	20.98	1.57	

Table S6. Constituents of the fraction F17 identified by Gas Chromatography Analysis (CPG).

Noun	Retention Time	Percentage	Structure
Alcohols			
3,5-Di-tert-butylphenol	16.23	3.95	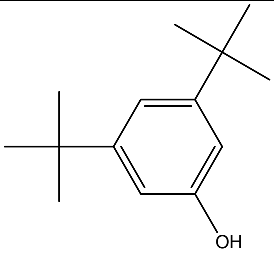
2-Heptanol, 5-ethyl	17.00	1.18	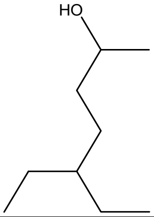
1H-Imidazole-2-methanol	14.00	0.67	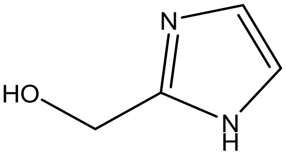
2-Pentadecyn-1-ol	20.74	3.05	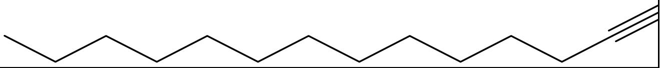
2<br>Hydroxy<br>Undecane	17.39	1.12	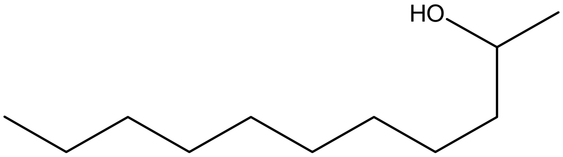
Cetone			
2-Undecanone, 6,10-dimethyl<br>cétone	18.68	9.68	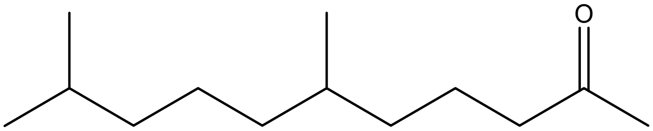
Esters			
Nicotinic acid, 1,6-dihydro-4 -hydroxy-2-methyl-6-oxo- ethyl ester ester	18.07	0.85	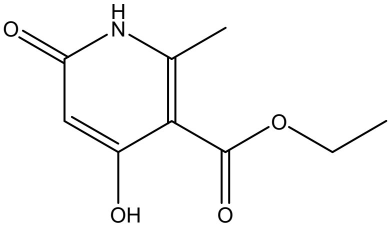
methacrylic acid octyl ester ester	17.21	0.86	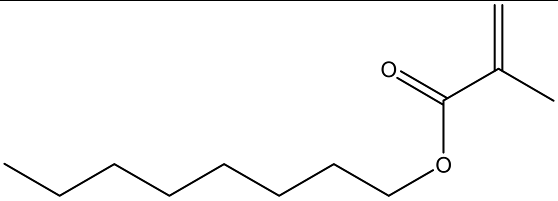

Hexadecanoic acid, methyl ester<br>ester	19.24	3.64	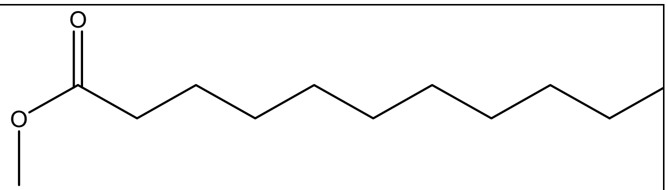
Ether			
2-Methoxytetrahydropyran<br>ether	15.06	1.27	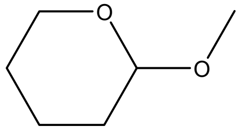
Unsaturated hydrocarbons			
9-Octadecene<br>Hydrocarbure insaturé	16.71	18.34	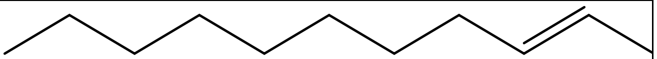
9-Octadecene, (E)<br>Hydrocarbure insaturé	18.22	18.61	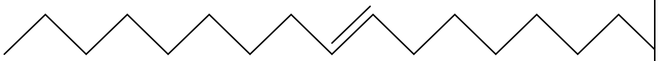
Saturated hydrocarbons			
Chl orooctadecane	17.72	0.98	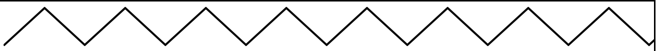
Others			
Acetic acid, bicy clo[2.2.1]hept-2-en-7-ylidene- Sydnone, 3-(4-nitrophenyl)-	19.02	3.46	
Acetic acid, bicy clo[2.2.1]hept-2-en-7-ylidene- 17-Pentatriacontene	19.76	8.35	
Silane, trichloroeicosyl-	21.54	1.02	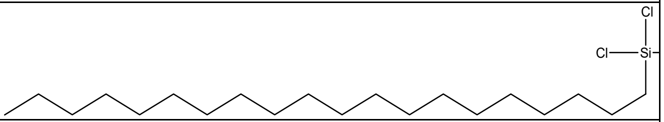
Isoxazole, 5-methyl-<br>(azole)	11.13	1.09	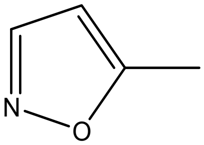
Methyl N-(3,4-dichlorophenyl) Carbamate<br>carbamate	19.58	3.11	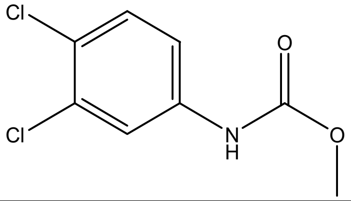
Propane, 2-isocyanato<br>cyanate	21.74	0.63	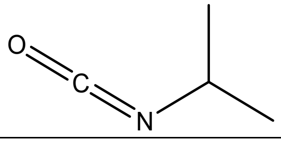

Table S7. Constituents of the fraction F18 identified by Gas Chromatography Analysis (CPG).

Noun	Retention Time	Percentage	structure
Alcohols			
**Hexahydropyridine, 4-[4,5-dihydroxyphenyl]**	**23.32**	**0.21**	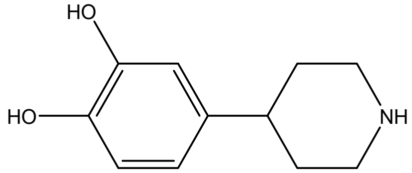
**3-Hydroxy -1-hexyne**	**15.07**	**0.35**	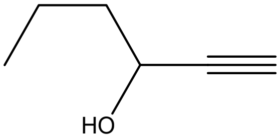
**1-Hexadecanol**	**20.73**	**2.75**	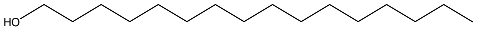
**3,7, 11,15-Tetramethylhexadecanol**	**20.44**	**1.79**	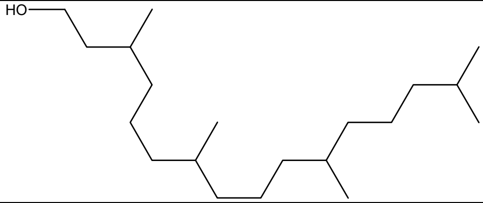
**1-Dodecanol, 2-methyl-, (S)-**	**21.54**	**1.10**	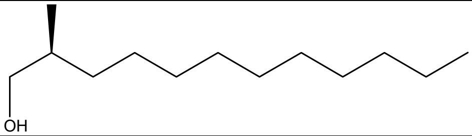
Aldehyde			
**4-hydroxytetradec-2-ynal**	**23.87**	**0.29**	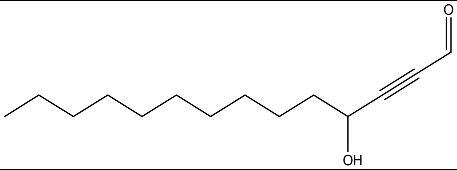
Amide			
**Propionamide (amides)**	**34,05**		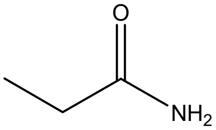
Cetones			
**5-Chloro-2-methyl-4-isothi azolin-3-one (cetone)**	**26.71**	**0.20**	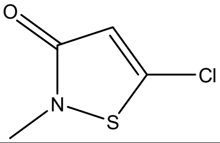
**1-(3,4)-Methylenedioxyphenyl-2-propanone oxime (cetone)**	**28.64**	**0.32**	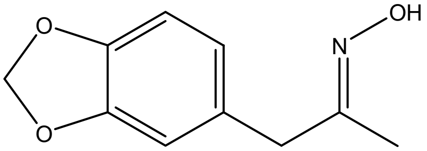
**Methyl n-hexyl ketone (Cétone)**	**18.67**	**8.95**	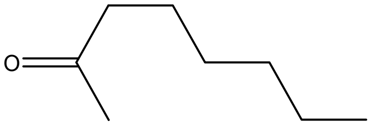
Amine			

**1,2-DIMETHYLAZETIDINE (Cycloamine)**	**11.15**	**0.35**	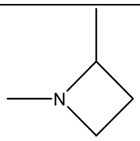
Esters			
**Heptanedioic acid, dimethyl ester (ester)**	**20.92**	**0.80**	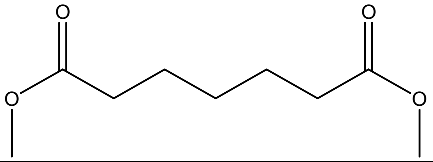
**Hexadecanoic acid, methyl ester (ester)**	**19.24**	**4.86**	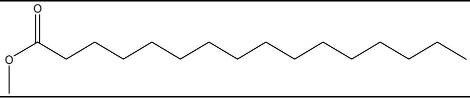
**Ethyl 2,2-Difluoro-4-iodooctanoate (esters)**	**35,24**	**0.35**	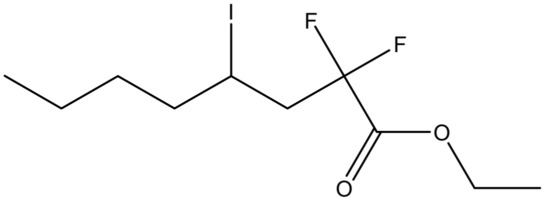
Saturated hydrocarbons			
**Eicosyltrichlorosilane (Hydrocaburesaturé)**	**19.77**	**10.24**	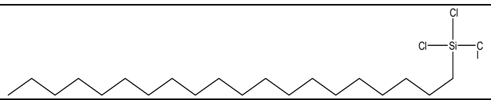
Others			
**(Z)-cis-6,7-Epoxyheptadec-9-ene (epoxy)**	**22.77**	**0.33**	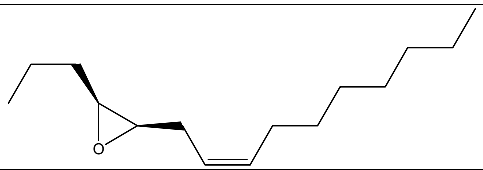
**CYANO METHYL-TRIIRON DISULFIDE OCT ACARBONYl**	**19.14**	**1.46**	
**2H-Benzotriazole, 2-ethyl**	**35,54**	**0.18**	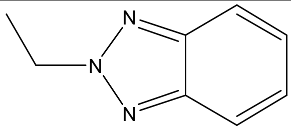
**1,6-dimethyl-pyrimido[5,4-e][1.2.4]triazine-5-one**	**28.43**	**0.23**	
**Bicycle(2.2.1)hept-2'-en-7'-ylideneacetic acid**	**20.06**	**0.98**	

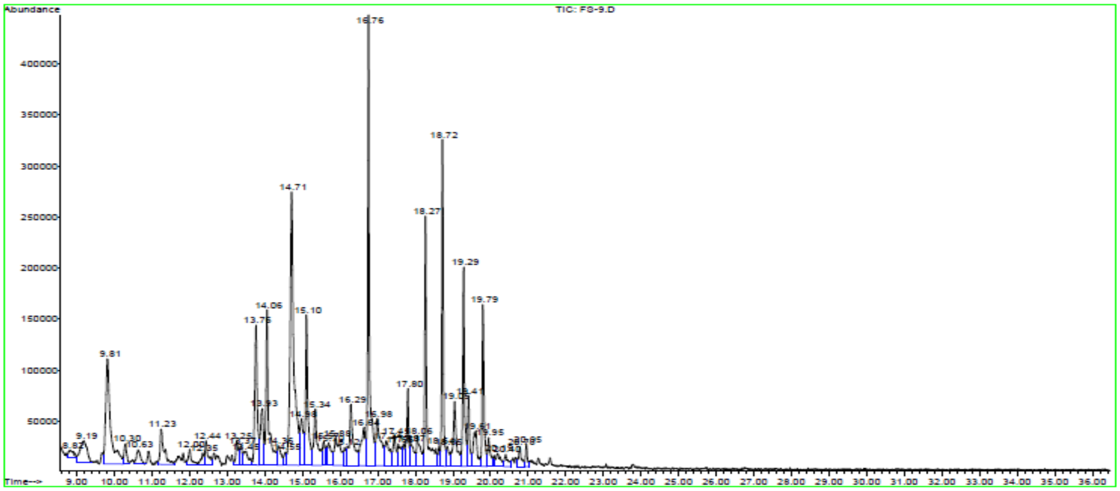

Figure S1. Chromatogram CPG of F9 dichloromethane extract fraction of *Raphanus raphanistrum* recorded on an apolar HP-5 column.

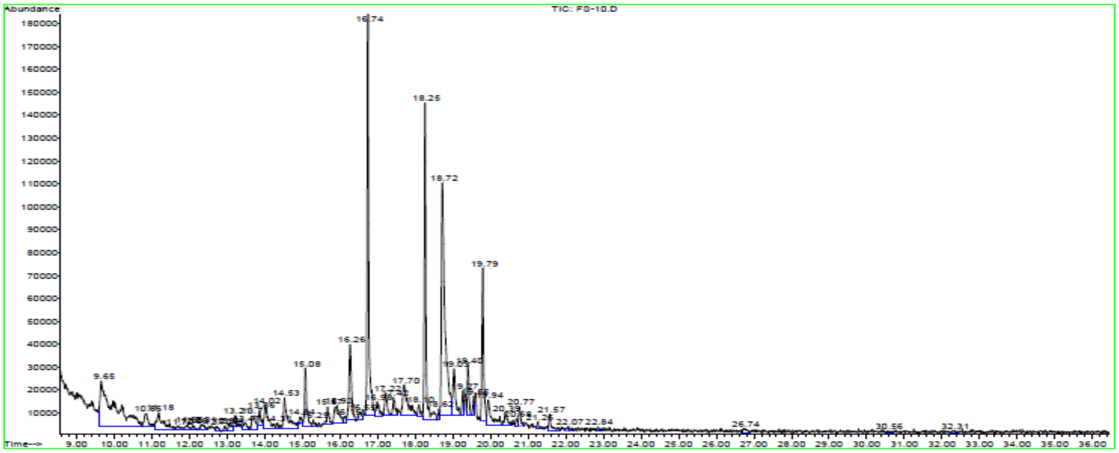

Figure S2. Chromatogram CPG of F10 dichloromethane extract fraction of *Raphanus raphanistrum* recorded on an apolar HP-5 column.

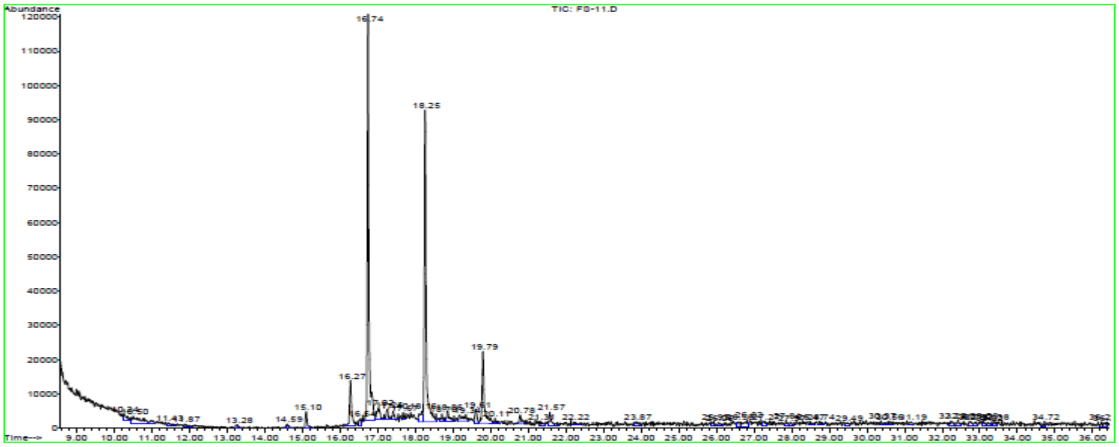

Figure S3. Chromatogram CPG of F11 dichloromethane extract fraction of *Raphanus raphanistrum* recorded on an apolar HP-5 column.

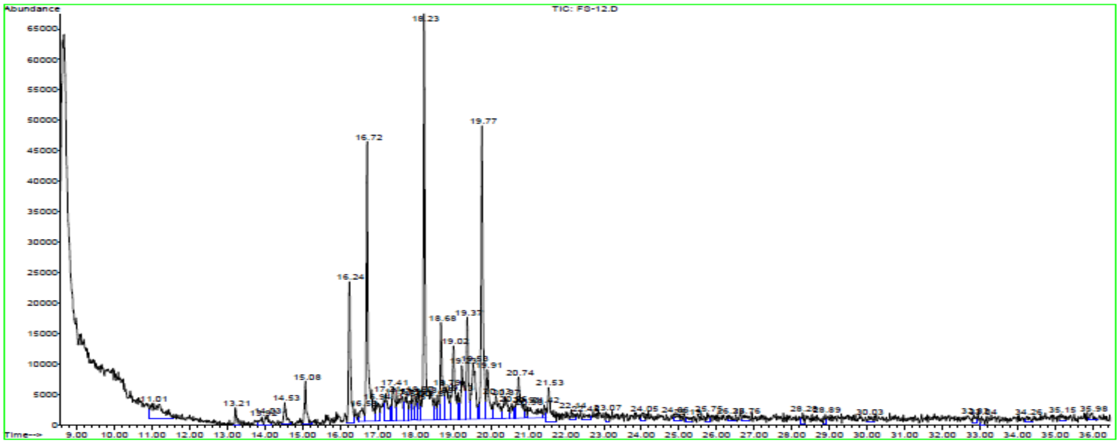

Figure S4. Chromatogram CPG of F12 dichloromethane extract fraction of *Raphanus raphanistrum* recorded on an apolar HP-5 column.

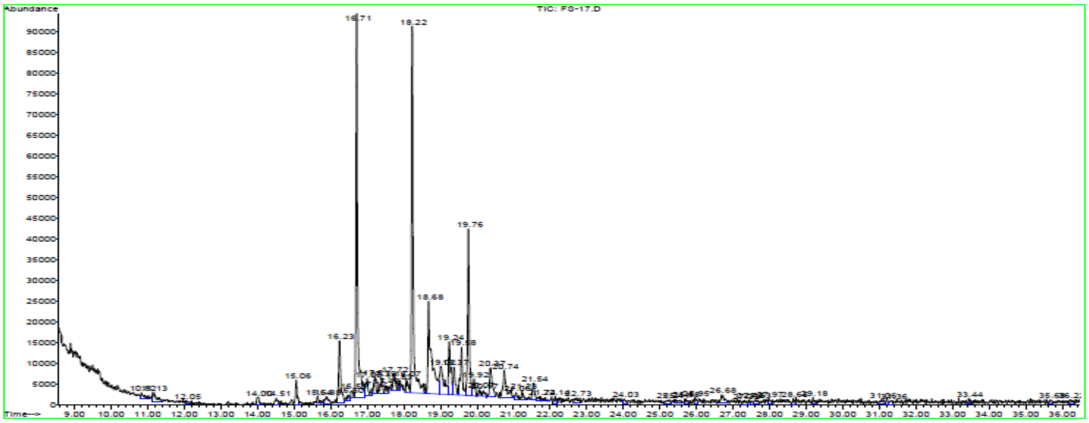

Figure S5. Chromatogram CPG of F17 dichloromethane extract fraction of *Raphanus raphanistrum* recorded on an apolar HP-5 column.

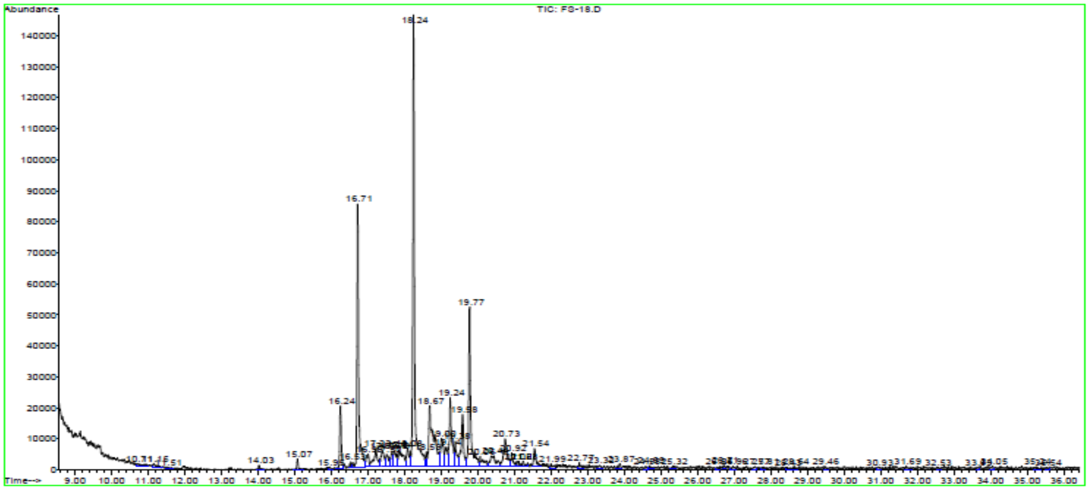

Figure S6. Chromatogram CPG of F18 dichloromethane extract fraction of *Raphanus raphanistrum* recorded on an apolar HP-5 column.
